# Methods to Detect Volatile Organic Compounds for Breath Biopsy Using Solid-Phase Microextraction and Gas Chromatography–Mass Spectrometry

**DOI:** 10.3390/molecules28114533

**Published:** 2023-06-03

**Authors:** Eray Schulz, Mark Woollam, Paul Grocki, Michael D. Davis, Mangilal Agarwal

**Affiliations:** 1 Department of Chemistry and Chemical Biology, Indiana University-Purdue University, Indianapolis, IN 46202, USA; erschulz@indiana.edu (E.S.); mwoollam@iu.edu (M.W.); 2 Integrated Nanosystems Development Institute, Indiana University-Purdue University, Indianapolis, IN 46202, USA; pgrocki@iu.edu; 3 Department of Pediatrics, Indiana University School of Medicine, Indianapolis, IN 46202, USA; mdd1@iu.edu; 4 Department of Mechanical & Energy Engineering, Indiana University-Purdue University, Indianapolis, IN 46202, USA

**Keywords:** volatile organic compound (VOC), direct-breath solid-phase microextraction (DB–SPME), Tedlar bags, gas chromatography-mass spectrometry quadrupole time-of-flight (GC–MS QTOF)

## Abstract

Volatile organic compounds (VOCs) are byproducts from metabolic pathways that can be detected in exhaled breath and have been reported as biomarkers for different diseases. The gold standard for analysis is gas chromatography–mass spectrometry (GC–MS), which can be coupled with various sampling methods. The current study aims to develop and compare different methods for sampling and preconcentrating VOCs using solid-phase microextraction (SPME). An in-house sampling method, direct-breath SPME (DB–SPME), was developed to directly extract VOCs from breath using a SPME fiber. The method was optimized by exploring different SPME types, the overall exhalation volume, and breath fractionation. DB–SPME was quantitatively compared to two alternative methods involving the collection of breath in a Tedlar bag. In one method, VOCs were directly extracted from the Tedlar bag (Tedlar–SPME) and in the other, the VOCs were cryothermally transferred from the Tedlar bag to a headspace vial (cryotransfer). The methods were verified and quantitatively compared using breath samples (*n* = 15 for each method respectively) analyzed by GC–MS quadrupole time-of-flight (QTOF) for compounds including but not limited to acetone, isoprene, toluene, limonene, and pinene. The cryotransfer method was the most sensitive, demonstrating the strongest signal for the majority of the VOCs detected in the exhaled breath samples. However, VOCs with low molecular weights, including acetone and isoprene, were detected with the highest sensitivity using the Tedlar–SPME. On the other hand, the DB–SPME was less sensitive, although it was rapid and had the lowest background GC–MS signal. Overall, the three breath-sampling methods can detect a wide variety of VOCs in breath. The cryotransfer method may be optimal when collecting a large number of samples using Tedlar bags, as it allows the long-term storage of VOCs at low temperatures (−80 °C), while Tedlar–SPME may be more effective when targeting relatively small VOCs. The DB-SPME method may be the most efficient when more immediate analyses and results are required.

## 1. Introduction

Volatile organic compounds (VOCs) are sources of potential biomarkers as they are products of metabolism and other biological processes that are uniquely dysregulated by different diseases and human conditions [1]. One technique used to detect VOC biomarkers is the training of canines to smell them in different biological sample types. Given their remarkable olfactory systems, canines can detect VOC biomarkers with high sensitivity and specificity. Currently, there are canines specialized to detect hypoglycemia, referred to as diabetes-alert dogs (DADs) [2,3]. Canines have also been shown to detect other diseases, such as lung cancer [4], prostate cancer [5], viral infections [6,7], and others with high accuracy. These results have inspired researchers to identify volatile biomarkers for different medical conditions, including type 1 diabetes [8], breast cancer [9], and lung cancer [10,11], using different analytical approaches. Integrated arrays using gas sensors (electronic noses, e-noses) provide rapid, portable, and easy-to-use devices that are capable of distinguishing disease states [12]. Nonetheless, these devices generally lack VOC-biomarker selectivity and utilize a black-box approach to disease detection [13,14]. The gold standard for VOC analysis is gas chromatography–mass spectrometry (GC–MS). The use of GC–MS can separate volatiles within a complex mixture, structurally elucidate analytes, and quantify them in biological samples. For example, VOC biomarkers have been detected in various sample types, including urine, sweat, blood, and exhaled breath [1,15]. The analysis of VOCs in exhaled-breath samples has gained significant attention over the years because it is noninvasive and nearly limitless in supply. Furthermore, the hundreds of VOCs in exhaled breath serve as a rich source of potential biomarkers [16].

Due to the low concentrations of VOCs in exhaled-breath samples, preconcentration techniques are often implemented prior to analysis [17]. A commonly performed method involves adsorption tubes, in which the breath sample is drawn over a sorbent bed (usually carbon-based materials or porous polymers, including Tenax) designed to trap VOCs [18]. These tubes can be stored at low temperatures, allowing long-term storage after breath sampling. One drawback of this specific preconcentration method is that VOCs must be introduced into the detection system using a relatively expensive thermal desorption unit [19,20]. An alternative preconcentration method is solid-phase microextraction (SPME). This is an off-column sampling method which utilizes polymer- and polymer-composite-coated microfibers to adsorb VOCs in samples [21]. Commercially produced SPME fibers are available with a wide range of coatings to adsorb volatiles varying in size, functionality, and polarity. Another commonly used and cost-effective tool is the needle-trap device (NTD). Comprised of a needle with a sorbent material similar to SPME fibers, breath VOCs can be preconcentrated for analysis using NTDs [22,23]. There are also commercially available sampling devices, such as the Rtube [24,25] and Tedlar bags [26,27], which are designed to collect breath samples. These methods are also capable of being coupled with preconcentration methods such as SPME. However, these sampling devices tend to be non-reusable and/or expensive. Moreover, differences in sampling methods across the literature lead to variations in VOC profiles [28,29]. Lastly, there have even been efforts aiming to integrate SPME devices into facemasks to more easily and directly extract VOCs expelled in human breath [30,31,32].

Along with various preconcentration and sampling methods, the standardization of the breath-collection method is vital given the variation in exhaled VOC profiles. For example, the rate at which a patient exhales can play a significant role in VOC expression. Differences in lung physiology, including capacity and exhalation duration, provide variables that lead to interpatient variability and, therefore, differences within and between studies [33]. These variables can be normalized between patients through the monitoring of exhaled-breath volume, exhalation rate, and carbon-dioxide levels with clinical capnographs [34,35]. With these factors in mind, the aim of the present study was to develop an in-house, easy-to-use, efficient, and cost-effective method for sampling VOCs in breath, termed direct-breath SPME (DB–SPME). Additionally, DB–SPME is compared to two other methods using Tedlar bags. One method is used to directly extract VOCs from breath collected in a Tedlar bag (Tedlar–SPME), and in the other method, breath VOCs are cryothermally transferred from a Tedlar bag to a headspace vial (cryotransfer). All the methods were optimized, standardized using a capnograph, and compared in terms of their parameters, including sensitivity and reproducibility.

## 2. Results

### 2.1. DB–SPME-Method Optimization

**Figure 1:**
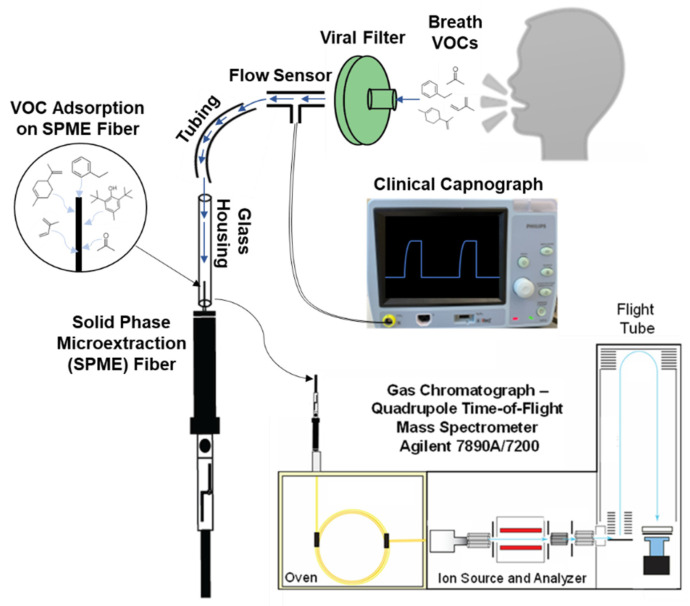
Illustration of the DB–SPME apparatus interfaced with a clinical capnograph, which allows CO_2_ and breath-volume measurements. The volunteer exhales downward through a viral filter onto a SPME fiber at the bottom of the system.

In order to maximize the VOC sensitivity for the DB–SPME method (illustrated in Figure 1), two SPME types (arrow and fiber) with various chemical compositions were tested, optimized, and compared. First, three SPME fiber-chemical compositions were analyzed: polydimethylsiloxane (PDMS), PDMS/carboxen (CAR), and PDMS/CAR/divinylbenzene (DVB). These SPME fibers were quantitatively compared regarding their overall GC–MS signal, as well as the number of VOCs detected. The PDMS/CAR/DVB SPME fiber not only had the ability to preconcentrate a significantly higher number of VOCs in exhaled breath via DB–SPME, but it also had the strongest overall GC–MS signal (Supplementary Figure S1). After optimizing the stationary phase of the SPME fiber, five SPME-arrow chemical compositions were also tested and compared using the DB–SPME method (polyacrylate, PDMS, PDMS/DVB, PDMS/Carbon Wide Range (CWR), and PDMS/CWR/DVB). Two PDMS arrows were tested: one was a standard arrow with a 100-μm coating thickness, and the other had a thicker stationary phase of 250 μm (PDMS 250). The results showed that the PDMS/CWR/DVB SPME arrow outperformed the other SPME-arrow compositions after the total GC–MS signal, the number of VOCs detected, and the signals of the individual VOCs were compared. The integrated signals for isoprene, limonene, and toluene for the different arrow compositions are shown in Supplementary Figure S2, displaying that the PDMS/CWR/DVB SPME arrow had the highest sensitivity.

**Figure 2:**
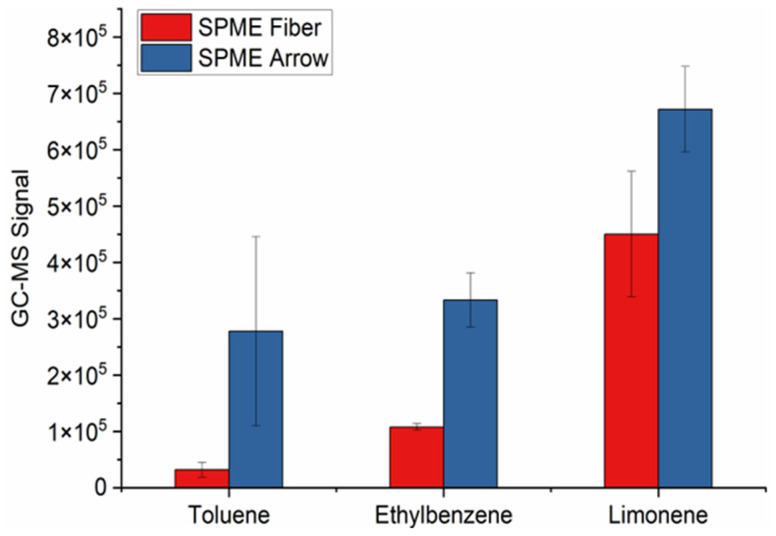
Bar plots illustrating the PDMS/CWR/DVB SPME arrow with greater sensitivity to toluene, ethylbenzene, and limonene than the PDMS/CAR/DVB SPME fiber when DB–SPME was performed.

**Figure 3:**
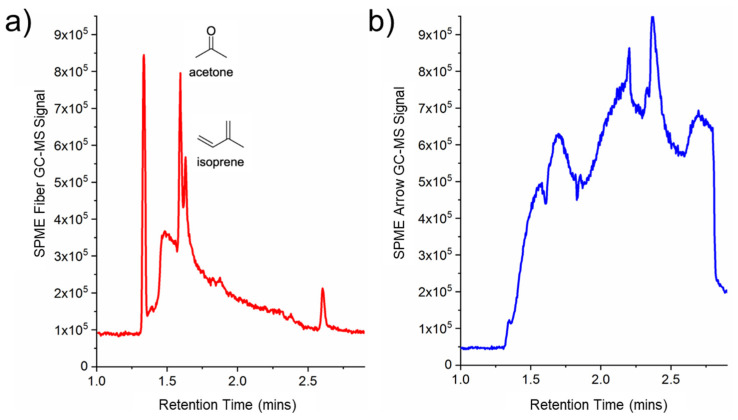
Chromatographic traces for VOCs with low molecular weight using the (**a**) SPME fiber and (**b**) SPME arrow, demonstrating the inability of the SPME arrow to detect acetone and isoprene.

After determining the optimal chemical composition for the SPME fiber and arrow, both were used to sample the exhaled VOCs using the DB–SPME method and were quantitatively compared. The PDMS/CWR/DVB SPME arrow demonstrated an increased sensitivity compared to the SPME fiber when the VOCs with relatively high molecular weights were analyzed. For example, Figure 2 illustrates increases in GC–MS signal for toluene, ethylbenzene, and limonene when utilizing the SPME arrow. However, the SPME arrow showed an increased susceptibility to releasing more thermal degradation products of the polymer-based stationary phase when compared to the SPME fiber (increased signals on GC–MS chromatograms for silanes and siloxanes). The SPME fiber, on the other hand, detected VOCs with lower molecular weights, such as acetone and isoprene, while the SPME arrow failed to detect these VOCs with high sensitivity. These results can be observed in the chromatographic traces of the SPME fiber (Figure 3a) and SPME arrow (Figure 3b), with the increased background signal of the SPME arrow also shown. Therefore, it was decided to proceed with the PDMS/CAR/DVB SPME fiber.

**Figure 4:**
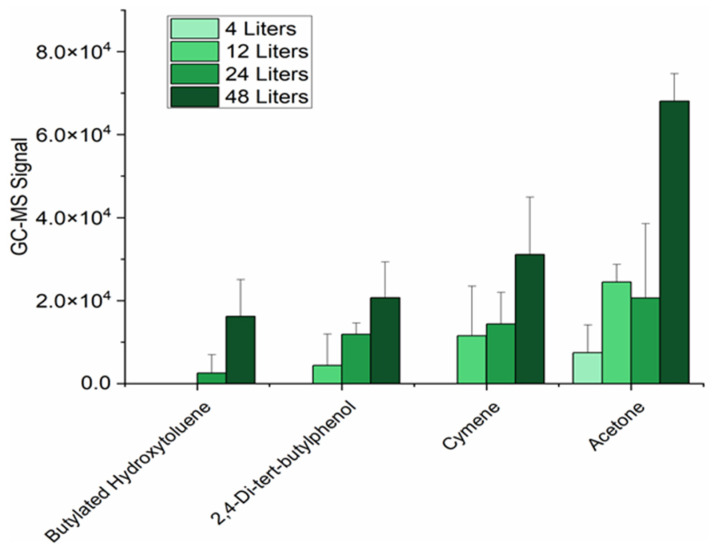
GC–MS signal of selected VOCs in breath with increased exhalation volume sampled using DB–SPME. As volume increased to 48 L, there were increases in method sensitivity.

Alternative parameters for DB–SPME were tested to develop a sensitive and comfortable method for sampling breath. First, different volumes of exhaled breath (4 L, 12 L, 24 L, and 48 L) were tested using DB–SPME with the optimal SPME fiber type. As the volume increased, the number of VOCs and the signals of individual VOCs significantly increased. The greatest sensitivity was observed when 48 L of breath was sampled by DB–SPME. For example, analytes such as acetone and cymene all demonstrated increases in signal that were positively correlated with exhalation volume (Figure 4). Therefore, optimization experiments proceeded with the sampling of 48 L of tidal breath. Next, the effect of breath fractionation was explored using the DB–SPME method by exposing the SPME fiber to whole-breath samples and alveolar breath samples. It is important to note that for the alveolar breath sampling, the SPME fiber was left unexposed until the CO_2_ concentration in the subject’s breath reached a plateau (monitored using a clinical capnograph). The alveolar breath did not show any significant increases in the number of VOCs detected or the total GC–MS signal (Supplementary Figure S3). Moreover, alveolar breath sampling via DB–SPME did not show any increases in VOC reproducibility when compared to the whole-breath samples. Therefore, the use of whole breath (non-fractionated breath) was deemed optimal, as it is an easier-to-deploy sampling method.

### 2.2. Tedlar–SPME-Method Optimization

**Figure 5:**
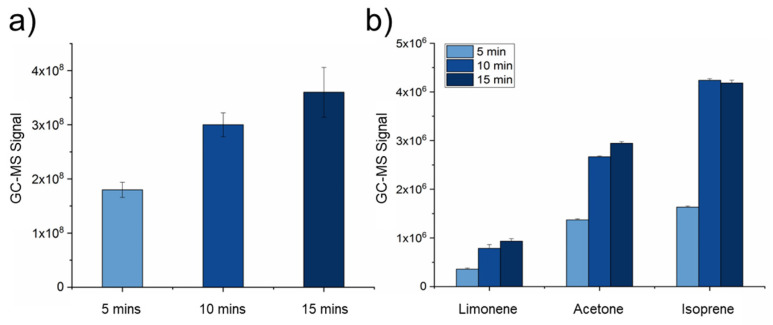
Bar plots showing the various SPME fiber-extraction times (5-, 10-, 15- minutes) that were explored using the Tedlar–SPME method. The 15-min condition yielded the greatest (**a**) total GC–MS signal and (**b**) individual VOC signals.

Once the DB–SPME VOC sampling technique was optimized, the extraction time for the Tedlar–SPME method was explored. To effectively preconcentrate VOCs from breath in a Tedlar bag, the extraction period in which a SPME fiber is exposed significantly affects the adsorption of exhaled VOCs. To explore this phenomenon, 5-, 10-, and 15-min incubation periods were tested by inserting the SPME fiber into the Tedlar bag through the septum after breath sampling, and the quantitative results demonstrated that the 15-min extraction time had the highest VOC sensitivity. For example, this method had a significantly greater total GC–MS integrated signal (Figure 5a). Individual VOCs including but not limited to those of limonene, acetone, and isoprene were also integrated and analyzed among the different extraction times. Although isoprene showed no significant differences between the 10- and 15-min extraction times, most of the VOCs showed stepwise increases in sensitivity when the extraction time was increased to 15 min (Figure 5b).

### 2.3. SPME-GC–MS-Method Comparison

**Figure 6:**
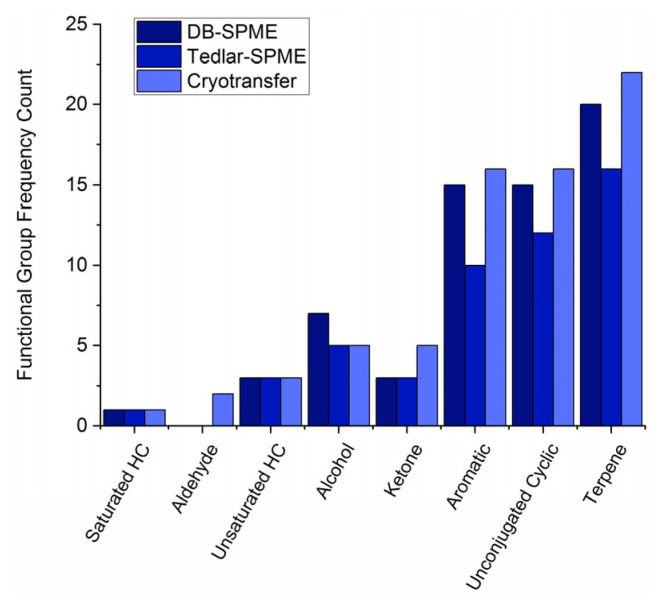
Bar plots of functional-group frequency for VOCs detected in at least one method (Tedlar–SPME, cryotransfer, or DB–SPME) and one of the three volunteers. Cryotransfer showed increased sensitivity in detection of most of the functional groups observed.

After the DB–SPME and Tedlar–SPME methods’ parameters were explored, these two sampling techniques were quantitatively compared to the previously optimized cryotransfer method [27]. Once the sample collection was complete, the VOCs were identified and tabulated for each volunteer using each breath-collection method. Exemplary GC–MS chromatograms from one of the volunteers are provided for each method in Supplementary Figure S4. To survey the VOCs as robustly as possible, the aim of our first screening procedure was to analyze compounds that were present in at least 80% of one method in at least one of the three volunteers. To represent these compounds, a functional-group-frequency analysis was undertaken for each of the methods independently (Figure 6). The detected functional groups were saturated and unsaturated hydrocarbons (HCs), aldehydes, alcohols, ketones, aromatics, unconjugated cyclics (nonaromatic cyclics), and terpenes. It can be observed that the terpenes, unconjugated cyclics, and aromatic compounds were the most frequently detected functional groups universally across all the methods. The saturated HCs, aldehydes, and unsaturated HCs, on the other hand, were the least frequently detected. The cryotransfer method displayed the ability to detect the highest number of VOCs for all the functional groups detected except for the alcohols, which were the most frequently detected using the DB–SPME method. The DB–SPME method generally had higher functional-group frequencies than the Tedlar–SPME for other features. There were limited-to-no differences between the methods’ abilities to detect saturated and unsaturated HCs.

**Figure 7:**
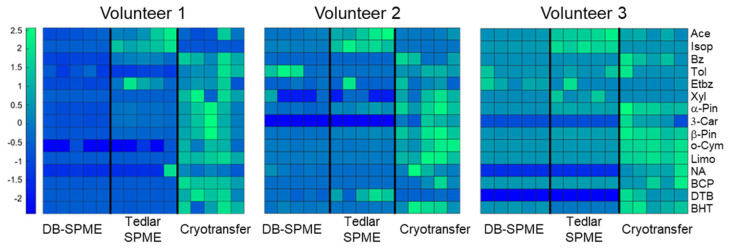
A heatmap illustrating the VOCs detected in each of the three methods (cryotransfer, DB–SPME, and Tedlar–SPME) across three different, relatively healthy volunteers. The cryotransfer method demonstrated greater sensitivity toward the majority of VOCs detected in breath compared to the DB–SPME and Tedlar–SPME methods.

Next, to further compare the relative performance of each method, the compounds were further filtered to only include the VOCs present in all three volunteers. The purpose was to compare each sampling method equally and quantitatively with respect to VOC sensitivity. Fifteen VOCs were identified in this limited set of analytes, and heatmaps for each volunteer were constructed (Figure 7). The VOCs are listed on the *y*-axis as rows, and the sample replicates are illustrated on the *x*-axis as columns. The VOC names are denoted within the heatmap using abbreviations (full compound names can be observed in Supplementary Table S1). Overall, the results were very consistent between the three volunteers, and they showed that the cryotransfer method was more sensitive to the majority of the identified compounds. These VOCs included but were not limited to butylated hydroxytoluene (BHT), limonene (Limo), o-cymene (o-Cym), 3-carene (3-Car), and other monocyclic terpenes. The aromatic VOCs, including benzene (Bz), toluene (Tol), ethylbenzene (Etbz), and xylene (Xyl), also tended to show stronger signals using the cryotransfer method. Although this trend of aromatic VOCs was not identified in all the volunteers, they were present in at least two of the volunteers. Isoprene and acetone, on the other hand, were most sensitively detected using the Tedlar–SPME method. The DB–SPME method generally presented the lowest sensitivity of the three methods.

**Figure 8:**
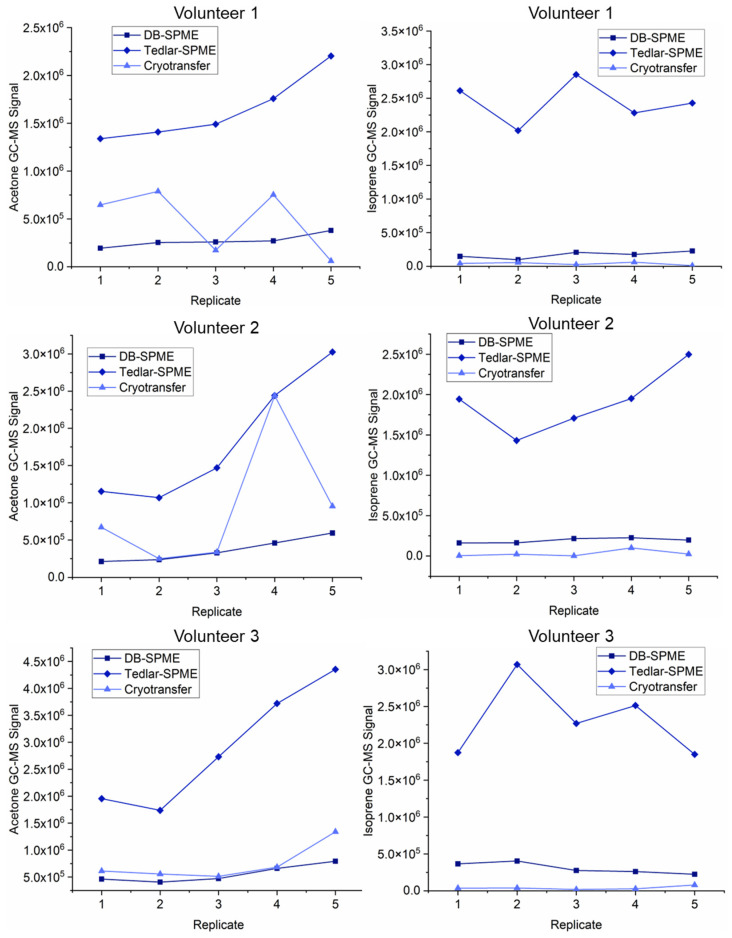
Scatter plots for acetone and isoprene for the three different methods across all three volunteers illustrate that Tedlar–SPME had greater sensitivity towards analytes with relatively low molecular weights.

**Figure 9:**
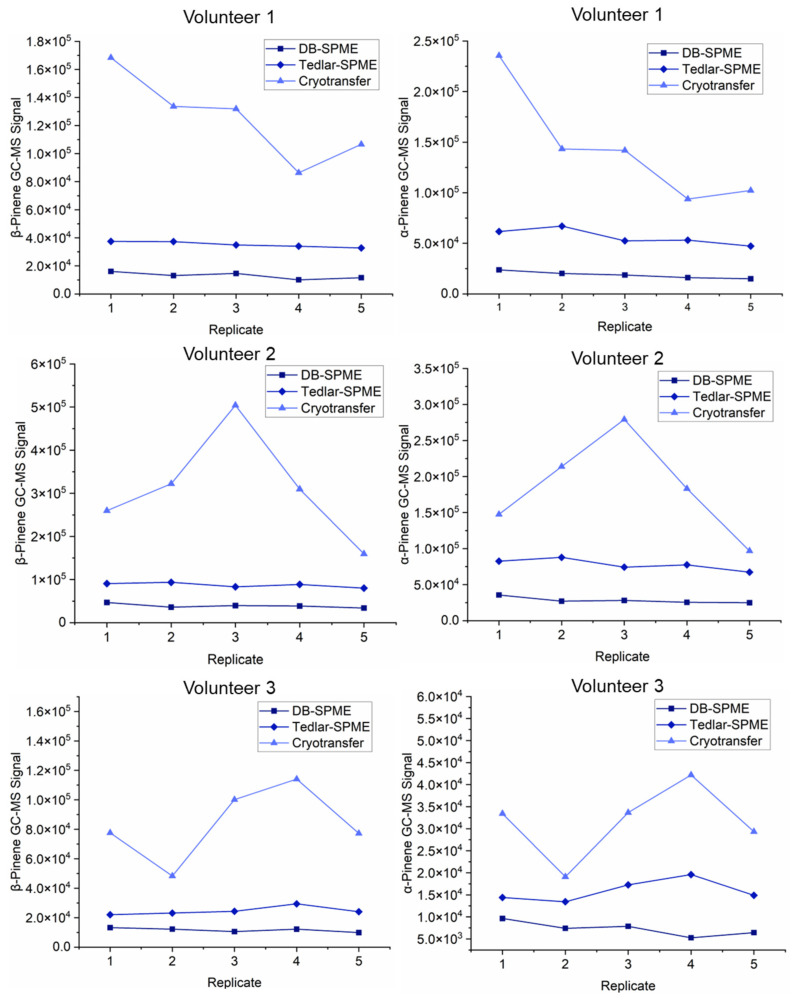
Scatter plots of pinene isomers for the three different methods across all three volunteers illustrate that cryotransfer had greater sensitivity towards analytes with higher molecular weights.

To better visualize the differences in the VOC trends between all three methods, scatterplots for the different methods across the three volunteers were generated. Figure 8 illustrates the background-subtracted GC–MS signals for acetone and isoprene, which were two of the analytes with the lowest molecular weights. The replicates on this graph are illustrated in chronological order of collection. The GC–MS signals for acetone and isoprene presented similar ranges between the three different volunteers. As initially depicted on the heatmap, these two compounds were consistently detected with the highest sensitivity using the Tedlar–SPME method across all the volunteers. The cryotransfer and DB–SPME methods had comparable abilities in the detection of acetone, and the DB–SPME generally had higher sensitivity for isoprene detection than the cryotransfer. It should be noted that the sample replicates within the cryotransfer method for acetone displayed higher variation within the volunteers when compared to the other two methods. Similar plots were generated for α- and β-pinene, which can be observed in Figure 9. The GC–MS sensitivity for these two VOCs was optimal when utilizing the cryotransfer method. These two compounds showed the same trends within the volunteers across the replicates, indicating a significant correlation in terms of signal. This is interesting, as these two VOCs are structural isomers.

**Figure 10:**
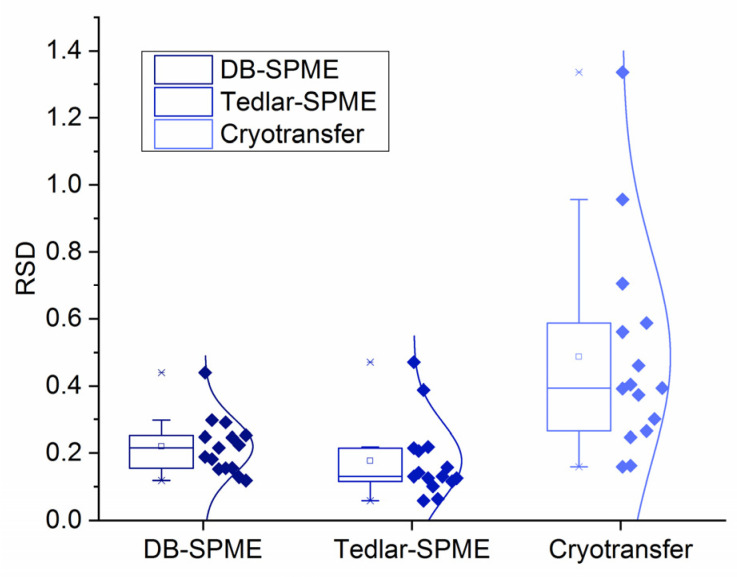
Relative standard deviation (RSD) values for VOCs detected consistently in all samples collected from all methods and volunteers showed cryotransfer had lower reproducibility than DB–SPME and Tedlar–SPME.

Lastly, to evaluate the reproducibility of each method, relative standard deviations (RSDs) were calculated for the VOCs detected in all 45 samples. This was performed to prevent RSD values from becoming skewed by the samples in which VOCs were not detected. Supplementary Table S2 shows the RSD values for five VOCs in each method and volunteer independently. To visually compare the RSD values between the different methods, VOC data from the different volunteers were compiled by method, and overlayed box and whisker plots were generated. The results are illustrated in Figure 10, which shows that the cryotransfer method had higher RSD values than the other two methods. DB–SPME and Tedlar–SPME, on the other hand, did not display significant differences regarding method reproducibility. For example, DB–SPME had RSD values ranging from 12–44%, and Tedlar–SPME had values between 6% and 47%. Cryotransfer, on the other hand, had values ranging from 16% to 134%. It should be noted that the RSD values for the cryotransfer were skewed by the lower-molecular-weight VOCs, including acetone (RSD range 46–96%) and isoprene (RSD range 56% to 134%).

## 3. Discussion

Given the unique characteristics of each sampling method, the experimental parameters were optimized individually prior to the comparison of their abilities to capture VOCs. All three methods were designed and optimized with the intention of performing the untargeted identification of VOCs in exhaled breath. The in-house DB–SPME method (illustration in Figure 1) was optimized by first testing various SPME-fiber and -arrow chemical compositions, as this plays a vital role in VOC sampling. For the SPME fiber, the PDMS/CAR/DVB stationary phase had the highest sensitivity (Supplementary Figure S1) because it detected a wide range of VOCs with different molecular weights and structures. The SPME arrow, on the other hand, showed that the PDMS/CWR/DVB coatings had the highest ability to adsorb VOCs (Supplementary Figure S2). The diverse chemical functionalities of both the PDMS/CAR/DVB fibers and the PDMS/CWR/DVB arrows is the hypothesized reason why they showed the highest sensitivity. Next, the optimal SPME fiber and arrow were compared directly, and the SPME arrow showed higher sensitivity to the VOCs with relatively high molecular weights, such as limonene and ethylbenzene (Figure 2). This is likely to have been due to the greater surface area of the SPME arrow relative to the SPME fiber. However, smaller VOCs, such as acetone and isoprene, remained undetected in the SPME arrow but were successfully sampled using the SPME fiber (Figure 3). The differences in VOC adsorption were expected, since CAR has a high ability to preconcentrate low-molecular-weight VOCs and CWR does not (SPME Fiber Coating Selection Guide, provided by Sigma Aldrich). Nonetheless, given the significance of these two VOCs (acetone and isoprene) as possible biomarkers [36,37,38], the SPME fiber was selected as optimal, and it was used for all three methods (DB–SPME, Tedlar–SPME, and cryotransfer). In summary, the presented results can help researchers to select SPME-fiber stationary phases that capture VOC biomarkers of interest with high sensitivity.

After determining the optimal SPME type and composition, the exhalation volume sampled by DB–SPME was optimized in a way that maintained the sensitivity to exhaled VOCs while remaining relatively comfortable for patients. When optimizing DB–SPME exhalation volume, it must be noted that the outlet diameter that serves as an interface between the SPME fiber and exhaled breath introduces the possibility of breath-dyssynchrony stacking (BDS) [39]. This occurs when incomplete exhalations between continuous breaths occur, resulting in breath mixing and higher tidal volumes. This can also lead to high intersubject variation when aiming to quantify and compare VOC profiles in multiple patients for VOC-biomarker-discovery applications, which is highly undesirable. Moreover, tidal breath sampling is the most widely used and accepted method for exhaled-VOC analysis [40,41]. Experiments using a narrow (2.00 mm) aperture diameter caused volunteers to feel resistance when exhaling, resulting in incomplete exhalations and BDS. Therefore, a wider (6.00 mm) diameter was utilized, which not only provided increased comfort when the volunteers exhaled, but also allowed tidal breathing and complete exhalations, thereby avoiding BDS. Increases in VOC sensitivity were observed when increasing tidal exhalation volume, and the method was shown to be optimal when sampling a total volume equal to 48 L (Figure 4). Although 48 L is a greater volume than that of a 3 L Tedlar bag, the time required for the breath sampling procedure was approximately five minutes, making this method comfortable and feasible for human-subject research.

Since exhaled breath consists of different phases [42], breath fractionation was also explored as a possible parameter to include when optimizing DB–SPME. By monitoring exhaled-CO_2_ profiles through capnography, the alveolar portion of breath can be detected and isolated. The SPME fiber was only exposed after this breath phase was reached (alveolar plateau), potentially reducing the adsorption of VOCs from the bronchial, tracheal, or oropharyngeal fractions onto the SPME fiber. Interestingly, the DB–SPME displayed no differences in sensitivity when the whole breath and fractionated alveolar breath were sampled (Supplementary Figure S3). As a result, the whole-breath sampling method was selected as it was a more straightforward method. For Tedlar–SPME, the optimization process placed an emphasis on the extraction time using a SPME fiber. Fifteen-minute extractions for Tedlar–SPME were selected, as it displayed significantly increased sensitivity (Figure 5). Due to the fact that samples in Tedlar bags have a storage time of 6 h, as reported in a previous study [43,44], increasing the incubation period for longer than 15 min may limit Tedlar–SPME in terms of the quantity of samples that can be collected and analyzed. Furthermore, although there was a slight increase in sensitivity between the 10-min and 15-min extraction times, there was also an observable plateau in the increases in VOC sensitivity for 15-min and beyond.

After optimizing the experimental parameters for DB–SPME and Tedlar–SPME, these two methods were compared to our previously optimized cryotransfer technique [45]. The cryotransfer method was previously utilized to successfully identify VOC biomarkers of hypoglycemia (low blood sugar) [45], clinical traits of cystic fibrosis [27], and COVID-19 [46]. For an accurate and robust comparison, three volunteers provided five replicates of breath for each of the three methods. Prior to the VOC quantitation, the functional-group frequency of VOCs in at least one of the methods and one of the volunteers was undertaken to visualize differences between the methods (Figure 6). The major functional groups detected in exhaled breath consisted of terpenes, nonaromatic cyclic compounds, aromatic VOCs, ketones, and other analytes. Terpenes/terpenoids are hypothesized to be endogenous metabolites biologically generated by the mevalonate pathway. This pathway is the precursor to the biosynthesis of cholesterol and has been found to be significantly altered by several medical conditions, including cancer [47]. Volatile carbonyls (ketones, aldehydes, etc.), on the other hand, are generated when cytochrome P450 (CYP450) reduces hydroperoxides through polyunsaturated-fatty-acid oxidation [1]. Increases in fatty-acid oxidation and oxidative stress are correlated with different medical diagnoses including but not limited to prostate cancer [48].

These analyses also showed that for most of the functional groups, the cryotransfer method detected the most VOCs. This initially indicated that the cryotransfer method was more sensitive than the others. To further explore this phenomenon, quantitative comparisons between the three methods among three different volunteers were undertaken. Here, heatmaps and scatter plots for the individual VOCs (Figure 7, Figure 8 and Figure 9) showed that cryotransfer was the most sensitive method for most of the VOCs, except for some of the analytes with lower molecular weights, including acetone and isoprene. For these low-molecular-weight VOCs, the Tedlar–SPME method was the most sensitive. However, cryotransfer showed the highest sensitivity to the VOCs with relatively high molecular weights, including but not limited to α-pinene, limonene, o-cymene, and other aromatic VOCs. These trends were observed in all three volunteers in the study. It should be noted that the scatter plots for α-pinene and β-pinene (Figure 9) showed similar trends within the volunteers, indicating a significant correlation between the levels of these two VOCs in exhaled breath. The fact that these two analytes are structural isomers indicates that there may be a relationship between VOC structure and expression in exhaled breath. This may also partly demonstrate the validity of the experimental results.

There are two possible reasons why the cryotransfer was not as sensitive to some of the low-molecular-weight VOCs. Relatively small analytes may be lost during the cryotransfer process due to the use of a vacuum, or larger VOCs could outcompete the low-molecular-weight VOCs to adsorb to the SPME fiber due to the extraction temperature of 60 °C used in this method. On the other hand, the rationale for why the cryotransfer method was the most sensitive to the other VOCs is two-fold. This method allows the concentration of VOCs in a 20-mL headspace vial, which is a much smaller volume than that of a 3 L Tedlar bag. This method also permits long-term storage and, therefore, the headspace vial can be agitated, heated, and extracted with a SPME fiber for a relatively long time of 45 min. The decreased sensitivity of the DB–SPME method is most likely to have been because the SPME fiber was exposed to the exhaled VOCs for a relatively short period of time. Nonetheless, because DB–SPME does not inherently rely on the collection of exhaled breath into a Tedlar bag, this method had the lowest background signal. In addition to sensitivity, method reproducibility was qualified by calculating the RSD values for five VOCs detected in all the samples across all the methods and volunteers (Figure 10 and Supplementary Table S2). These analyses show that the cryotransfer method had elevated RSD values, but this was mostly due to the heterogeneity of the low-molecular-weight VOCs (acetone and isoprene), which the method itself has more difficulty detecting.

Despite their differences in sample collection and VOC sensitivity, each of the three methods provide a unique set of characteristics that may be optimal, depending on the sampling environment and the requirements of the analysis. For example, DB–SPME allows rapid and straightforward sample collection, which may be applicable in point-of-care settings that involve samples which require more immediate analysis and results. Moreover, because of the relatively low background signal, VOC biomarkers significantly upregulated by different diseases may be detected using DB–SPME. Currently, there is no standard method for breath analysis in point-of-care settings, but future work utilizing DB–SPME and portable GC–MS systems may allow rapid analysis in the field. The other two methods (Tedlar–SPME and cryotransfer) rely on the use of Tedlar bags and, therefore, may be used in more experimental or discovery-based VOC investigations. These methods allow exhaled breath collection in the field, where access to analytical instrumentation, such as a GC–MS, is limited. These two methods may be used in conditions that do not require immediate VOC analysis, as Tedlar bags provide a means of storage. Tedlar–SPME may be used when samples can be efficiently analyzed by GC–MS within a 6-h window, especially in applications in which low-molecular-weight VOCs, such as acetone and isoprene, are of interest. On the other hand, cryotransfer may be used for untargeted VOC-biomarker investigations in which many samples are collected in the field that cannot be analyzed by GC–MS within a 6-h window, as cryotransfer is the most sensitive method and, in general, provides longer storage times in freezers at −80 °C. Although we do not report the specific lifetimes of cryothermally transferred samples, further studies may be undertaken to quantify an accurate timespan over which samples can be stored.

We suggest that due to the rapid nature of DB–SPME and cryotransfer’s high sensitivity, combined with the ability it offers to store samples, these are the two optimal methods for breath analysis using SPME GC–MS. The limitations of this study include the fact that breath samples from healthy volunteers were used to compare the three different sampling methods (DB–SPME, Tedlar–SPME, and cryotransfer). Therefore, the methods’ sensitivity to other VOCs, including significant biomarkers of different human conditions, were not assessed directly. In addition, the current study did not compare the three sampling methods based on SPME to the performance of adsorption tubes, another common method for preconcentrating exhaled VOCs [49]. Nonetheless, the results show that an in-house, rapid, and simple sampling technique (DB–SPME) can be used to detect VOCs expressed in breath noninvasively, such as previously identified biomarkers of different diseases, including but not limited to isoprene, acetone, and limonene [37,50,51,52,53,54]. Furthermore, the DB–SPME method was compared to two alternative methods of VOC analysis using Tedlar bags (Tedlar–SPME and cryotransfer), and it was shown that all three sampling methods have different benefits and may be used depending on the application of interest.

## 4. Materials and Methods

### 4.1. Materials and Instrumentation

Three-liter Tedlar gas sampling bags were used to collect exhaled breath and were purchased from Restek (Bellefonte, PA, USA). An MCS-series mass-flow controller purchased from Alicat Scientific (Marana, AZ, USA) was used to establish vacuum for the cryothermal transfer of breath. Fourteen-gauge hypodermic stainless-steel needles (Med-Vet International; Mettawa, IL, USA), deactivated glass wool, and 20-mL headspace vials with magnetic screw-thread headspace caps (Restek) were used in the cryotransfer process. ViroMax viral and bacterial filters (A-M Systems; Sequim, WA, USA) were used to remove any viral or bacterial agents from breath samples. A Philips NM3 capnograph (Murrysville, PA, USA) was acquired and used to monitor carbon-dioxide levels and exhalation volumes. Glass Pasteur pipettes used for DB–SPME were purchased from Fisher Scientific (Hampton, NH, USA). A 7890A GC coupled with a 7200-quadrupole time-of-flight (QTOF) MS manufactured by Agilent (Santa Clara, CA, USA) with a PAL autosampler (CTC Analytics; Raleigh, NC, USA) was used to analyze VOCs. A Restek Rxi-5ms GC column 30 m in length, 0.25 mm in internal diameter, and with a 0.25-μm film thickness was used to separate VOCs. A two-centimeter PDMS/CAR/DVB SPME fiber from Supelco (Bellefonte, PA, USA) and a PDMS/CWR/DVB SPME arrow from Restek were used for VOC preconcentration. High-density polyethylene (HDPE) was utilized to monitor instrumental variation throughout experiments.

### 4.2. DB-SPME Method Optimization

The DB–SPME method is designed to directly extract VOCs from exhaled breath using a SPME fiber or arrow (Figure 1). Subjects breathe through the viral filter at the top of the apparatus, which is interfaced with the clinical capnograph to allow monitoring of exhaled CO_2_ profiles and breath volume. The viral filter is connected using Tygon tubing to a glass pipette, where the SPME fiber is housed and exposed to VOCs in breath. As subjects breathe down into the apparatus, VOCs adsorb to the SPME fiber/arrow. The outlet of the pipette where the SPME fiber is housed was relatively wide (6.00 mm) to allow for tidal breath sampling. The following parameters were surveyed when optimizing the method: SPME type/chemical composition, overall exhalation volume, and breath fractionation. For SPME optimization, fibers and arrows with various chemical compositions were tested. These included SPME fibers coated with PDMS/CAR/DVB, PDMS/CAR, and PDMS. In addition, SPME arrows coated with polyacrylate, PDMS, PDMS/DVB, PDMS/CWR, and PDMS/CWR/DVB were also tested. Overall exhalation volume (measured using the clinical capnograph) was tested by sampling 4 L, 12 L, 24 L, and 48 L of breath. Breath fractionation was explored by monitoring CO_2_ levels via the clinical capnograph to identify differences in exhaled VOCs between alveolar (fractionated) and whole (non-fractionated) breath. To sample alveolar VOCs, the SPME fiber was left retracted until the exhaled CO_2_ concentration reached a plateau. Five replicates of breath samples were analyzed for each condition for all the optimization experiments.

### 4.3. Tedlar–SPME and Cryotransfer Optimization

The Tedlar–SPME method is used for short-term storage; VOCs in Tedlar bags vary in stability, ranging from 6 hours up to 7 days, depending on the size, volatility, and amount of humidity in the bag [43]. The cryotransfer method, on the other hand, is more suitable for long-term storage, as headspace vials containing breath VOCs can be stored at −80 °C. For both methods, 3 L Tedlar bags were purged with ultra-high-purity nitrogen for two minutes prior to sampling. When each volunteer was ready, the Tedlar bag was filled to approximately 80% capacity by exhaling through a viral filter interfaced with a clinical capnograph and the inlet of the bag. The SPME fiber/arrow identified in the DB–SPME optimization experiments was used for both Tedlar–SPME and cryotransfer. For extracting exhaled VOCs using Tedlar–SPME at room temperature, different incubation times were tested (5-, 10-, and 15-min, *n* = 5) with the SPME fiber/arrow and chosen based on previously reported intervals [55,56]. For cryotransfer, a previously optimized technique was used to transfer exhaled VOCs collected in the bag into 20-mL headspace vials loaded with 0.125 g of deactivated glass wool [45,46]. These vials were cooled for 15 min with dry ice prior to VOC transfer. Two 14-gauge needles were inserted through the cap of the vial, with one connected to the bag and the other to a mass-flow controller to establish vacuum. Next, the valve of the Tedlar bag was opened, allowing the vacuum to draw VOCs into the vial, where they adsorbed to the inner walls and the glass wool. Once the bags were empty, the vials were stored in a freezer at −80 °C. The vials were defrosted, and VOCs were extracted using the optimal SPME fiber/arrow for 45 min, while the sample was agitated at 250 RPM and heated to 60 °C. The SPME assembly was then injected into the inlet of the GC–MS system for analysis.

### 4.4. Comparison of DB–SPME, Tedlar–SPME, and Cryotransfer Methods

For the comparison of all three methods (DB–SPME, Tedlar–SPME, and cryotransfer), three volunteers provided breath samples on the same day to minimize any variations in breath VOCs. Sample collection started in the morning, prior to the consumption of any foods or beverages, excluding water. Fifteen replicates of each method were performed (*n* = 5 for three volunteers; *n* = 45 in total), with background samples collected prior to and after breath sampling. To further minimize VOC variation, the volunteers interpolated samples by first performing DB–SPME and then filling two Tedlar bags; one bag was used for Tedlar–SPME and the other for cryotransfer. Tedlar–SPME samples were extracted immediately, and cryotransfer samples were transferred within 30 min after initial collection. The HDPE standards were run each day of the analysis to track any variations in instrument performance and results.

### 4.5. SPME GC-MS Protocol and Data Analysis

The SPME fibers/arrows were pre-conditioned for 30 min prior to the first extraction and daily for 10 min at 250 °C. After extraction (using DB–SPME, Tedlar–SPME or cryotransfer), the SPME fiber/arrow was injected into the inlet of the GC–MS QTOF for four-and-a-half minutes to thermally desorb VOCs into the system. The method consisted of maintaining the oven temperature for two minutes at 40 °C followed by a ramp to 100 °C at a rate of 8 °C/min, a 15 °C/min ramp to 120 °C, 8 °C/min to 180 °C, 15 °C/min to 200 °C, and a final ramp of 8 °C/min to 260 °C. Ultra-high-purity helium was used as the mobile phase at a flow rate of 1.2 mL/min. The mass analyzer was implemented in full scan mode, ranging from 26 to 400 *m*/*z*. The ion source operated at 250 °C with an emission current of 4 µA. Agilent MassHunter was used to collect data in centroid format. Agilent MassHunter Navigator software was used to analyze VOCs through integrated signals. The GC–MS signals were background-subtracted and compared for method optimization and comparison. Here, ion chromatograms were extracted for the base *m/z* peak corresponding to analytes in breath and background samples. For the comparison of methods, VOCs detected in at least 80% of one method within at least one of the volunteers (after background subtraction) were first surveyed and identified through mass spectral matching using the NIST17 library, as well as by comparing the compound’s nonpolar retention index (NPRI) in NIST to an experimental NPRI calculated by an instrument-specific calibration curve [9]. Within this data set, functional-group-frequency analysis was undertaken after identification. For quantitative assessment, VOCs were limited to analytes identified in all three volunteers, and heatmaps were generated. Scatter plots for individual VOCs were also constructed to further visualize the differences in sensitivity among the methods. Lastly, reproducibility was assessed through calculating RSD values for VOCs detected in all three methods and volunteers.

## 5. Conclusions

The current study sought to develop a standardized method to directly sample exhaled VOCs (DB–SPME) and analyze them by GC–MS. The DB–SPME method was quantitatively compared to two other breath-sampling methods (cryotransfer and Tedlar–SPME), and it was shown that the cryotransfer method was the most sensitive to the majority of the VOCs detected, with the exception of VOCs with low molecular weights, which were most effectively detected using the Tedlar–SPME method. On the other hand, DB–SPME offered benefits such as reduced background and the elimination of VOCs originating from the Tedlar bags. Overall, DB–SPME may be more efficient when a relatively rapid analysis is required, while the cryotransfer method may be more suitable when large numbers of samples are collected in the field. Tedlar–SPME may be the most effective when targeting smaller VOCs for different applications. The comparison between and characteristics of each method constitute a step towards the standardization of a universal method for VOC analysis through SPME GC–MS. Future work will include the development of a system that mimics exhalation, thereby allowing the accurate quantification of VOCs in breath. Additionally, the current research can be leveraged in future investigations focusing on VOC-biomarker discovery. For example, further research could select a breath-sampling method that would be appropriate for the desired application based on the results presented in this study.

## Data Availability

Data presented in this study are available upon request from the corresponding author.

## Supplementary Materials

The following supporting information can be downloaded at: https://www.mdpi.com/article/10.3390/molecules28114533/s1. Table S1. List of VOCs illustrated on the heatmap (Figure 7) and their associated abbreviations, retention times, base peaks, and calculated nonpolar retention indices; Table S2. Relative standard deviation (RSD) values for VOCs detected in all the methods among all volunteers; Figure S1. Bar charts demonstrating the differences in (a) number of VOCs detected and (b) total GC–MS signal for SPME fibers with different chemical compositions (PDMS, PDMS/CAR and PDMS/CAR/DVB). The PDMS/CAR/DVB SPME fiber displayed the greatest ability to adsorb VOCs in a breath sample using DB–SPME; Figure S2. Bar plots illustrating the GC–MS signals of (a) isoprene, (b) limonene, and (c) toluene detected by DB–SPME using a SPME arrow with various chemical compositions. The PDMS/CWR/DVB arrow displayed the greatest ability to extract the highlighted VOCs; Figure S3. Bar plots illustrating the (a) number of VOCs and (b) GC–MS signal for fractionated (alveolar) and whole-breath samples analyzed using DB–SPME. No differences in sensitivity or reproducibility were observed and, therefore, whole-breath sampling was selected as it reduces the complexity of the DB–SPME method. Figure S4. Sample GC–MS chromatograms for each of the three methods when used to sample and detect exhaled VOCs from one of the volunteers.
