# Supplementary material for: Methods to Detect Volatile Organic Compounds for Breath Biopsy Using Solid-Phase Microextraction and Gas Chromatography–Mass Spectrometry
Source: Molecules. 2023 Jun 3;28(11):4533. doi: 10.3390/molecules28114533 (PMC10254745; doi:10.3390/molecules28114533)
Supplement: Supplementary file 1 [file molecules-28-04533-s001.zip › molecules-2414516-supplementary.pdf]

Supplementary Information

Methods to Detect Volatile Organic Compounds for Breath Biopsy Using Solid-Phase Microextraction and Gas Chromatography–Mass Spectrometry

Eray Schulz [1,2], Mark Woollam [1,2], Paul Grocki [2], Michael D. Davis [3] and Mangilal Agarwal [1,2,4,*]

[1] Department of Chemistry and Chemical Biology, Indiana University-Purdue University, Indianapolis, IN 46202, USA; erschulz@indiana.edu (E.S.); mwoollam@iu.edu (M.W.)
[2] Integrated Nanosystems Development Institute, Indiana University-Purdue University, Indianapolis, IN 46202, USA; pgrocki@iu.edu
[3] Department of Pediatrics, Indiana University School of Medicine, Indianapolis, IN 46202, USA; mdd1@iu.edu
[4] Department of Mechanical & Energy Engineering, Indiana University-Purdue University, Indianapolis, IN 46202, USA
* Correspondence: agarwal@iupui.edu

Table of Contents

Table S1: List of VOCs illustrated in the heatmap (Figure 7) and their associated abbreviations, retention times, base peaks, and calculated nonpolar retention indices.

Table S2: Relative standard deviation (RSD) values for VOCs detected in all the methods among all volunteers.

Figure S1: Bar charts demonstrating the differences in (a) number of VOCs detected as well as (b) total GC-MS signal for SPME fibers with different chemical compositions (PDMS, PDMS/CAR and PDMS/CAR/DVB). The PDMS/CAR/DVB SPME fiber displayed the greatest ability to adsorb VOCs in a breath sample using DB-SPME.

Figure S2: Bar plots illustrating the GC-MS signals of (a) isoprene, (b) limonene, and (c) toluene detected by DB-SPME using a SPME arrow with various chemical compositions. The PDMS/CWR/DVB arrow displayed the greatest ability to extract the highlighted VOCs.

Figure S3: Bar plots illustrating the (a) number of VOCs and (b) GC-MS signal for fractionated (alveolar) and whole breath samples analyzed using DB-SPME. No difference in sensitivity or reproducibility was observed and therefore, whole breath sampling was selected as it reduces the complexity of the DB-SPME method.

Figure S4: Sample GC-MS chromatograms for each of the three methods when used to sample and detect exhaled VOCs from one of the volunteers.

Table S1. List of VOCs illustrated in the heatmap (Figure 7) and their associated abbreviations, retention times, base peaks, and calculated nonpolar retention indices.

VOC Name	Abbreviation	Retention Time (mins)	Base Peak (m/z)	Nonpolar Retention Index (NPRI)
**Acetone**	Ace	1.6	43	551.3
**Isoprene**	Isop	1.7	67	556.3
**Benzene**	Bz	2.6	78	613.4
**Toluene**	Tol	4.1	91	706.6
**Ethylbenzene**	Etbz	6.0	91	822.7
**Xylene**	Xyl	6.1	91	832.0
**α-Pinene**	α-Pin	7.5	93	917.7
**3-Carene**	3-Car	7.8	93	936.3
**β-Pinene**	β-Pin	8.4	93	973.6
**o-Cymene**	o-Cym	9.4	119	1035.0
**Limonene**	Limo	9.5	67	1041.9
**Nonanal**	NA	10.9	57	1126.0
**(E)- β-caryophyllene**	BCP	15.8	91	1433.0
**2,4-Ditert-butylphenol**	DTB	17.0	191	1506.3
**Butylated hydroxytoluene**	BHT	17.1	205	1510.6

Table S2. Relative standard deviation (RSD) values for VOCs detected in all the methods among all volunteers.

	**DB-SPME RSD**			**Tedlar-SPME RSD**			**Cryotransfer RSD**		
	V1	V2	V3	V1	V2	V3	V1	V2	V3
**Acetone**	0.25	0.44	0.29	0.21	0.47	0.39	0.71	0.96	0.46
**Isoprene**	0.30	0.15	0.25	0.13	0.21	0.22	0.56	1.34	0.59
**α-Pinene**	0.19	0.15	0.22	0.14	0.10	0.16	0.39	0.37	0.27
**β-Pinene**	0.18	0.13	0.12	0.06	0.06	0.12	0.25	0.40	0.30
**Limonene**	0.15	0.22	0.25	0.12	0.13	0.12	0.16	0.16	0.39

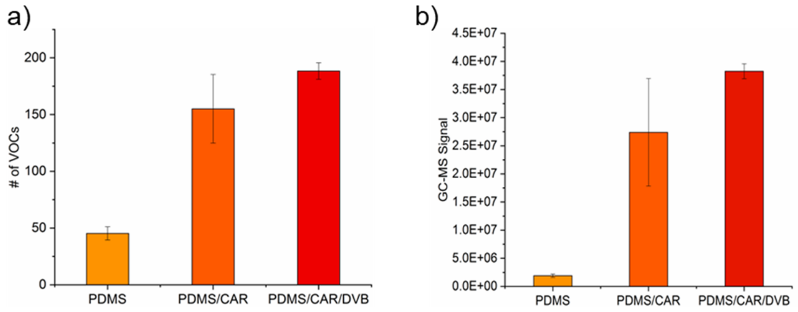

Figure S1. Bar charts demonstrating the differences in (a) number of VOCs detected as well as (b) total GC-MS signal for SPME fibers with different chemical compositions (PDMS, PDMS/CAR and PDMS/CAR/DVB). The PDMS/CAR/DVB SPME fiber displayed the greatest ability to adsorb VOCs in a breath sample using DB-SPME.

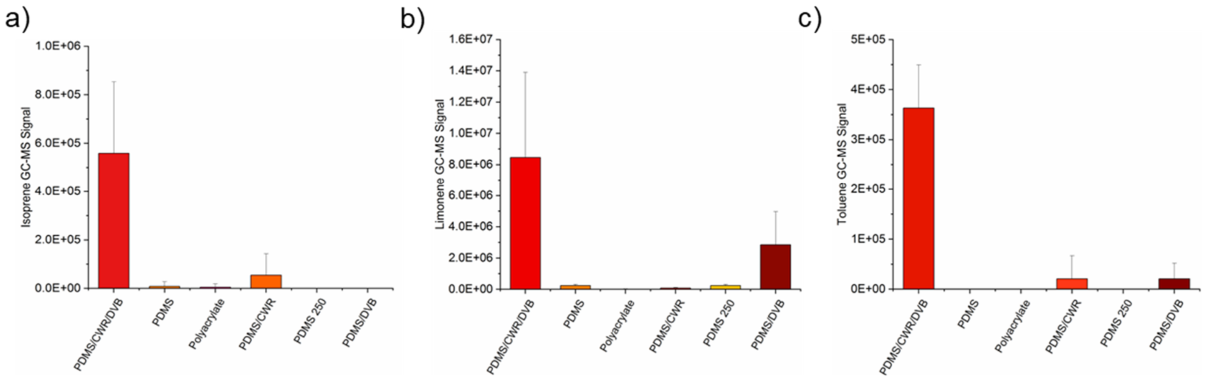

Figure S2. Bar plots illustrating the GC-MS signals of (a) isoprene, (b) limonene, and (c) toluene detected by DB-SPME using a SPME arrow with various chemical compositions. The PDMS/CWR/DVB arrow displayed the greatest ability to extract the highlighted VOCs.

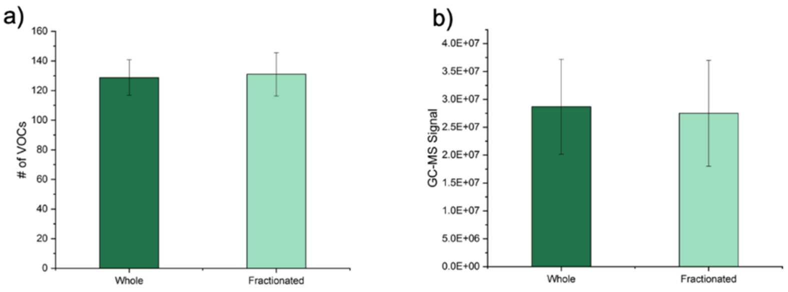

Figure S3. Bar plots illustrating the (a) number of VOCs and (b) GC-MS signal for fractionated (alveolar) and whole breath samples analyzed using DB-SPME. No difference in sensitivity or reproducibility was observed and therefore, whole breath sampling was selected as it reduces the complexity of the DB-SPME method.

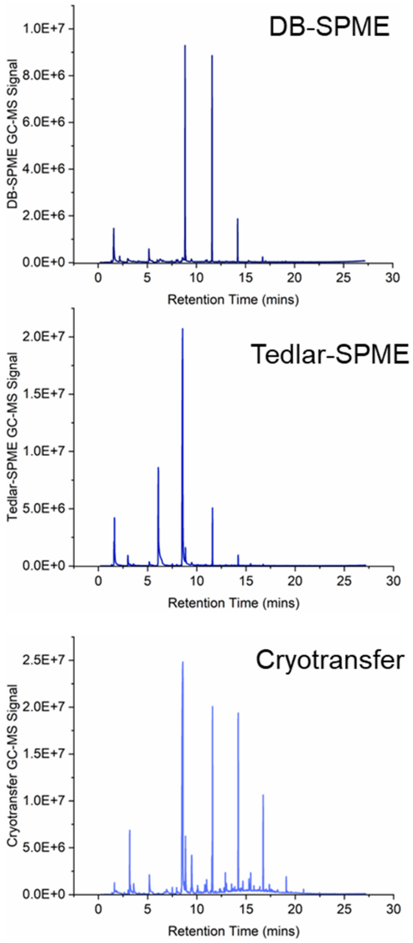

Figure S4. Sample GC-MS chromatograms for each of the three methods when used to sample and detect exhaled VOCs from one of the volunteers.
